# Wnt signaling mediates new nephron formation during zebrafish kidney regeneration

**DOI:** 10.1242/dev.168294

**Published:** 2019-04-29

**Authors:** Caramai N. Kamei, Thomas F. Gallegos, Yan Liu, Neil Hukriede, Iain A. Drummond

**Affiliations:** 1 Massachusetts General Hospital, Department of Medicine, Nephrology Division, 149 13th Street, Charlestown, MA 02129, USA; 2 Department of Developmental Biology, University of Pittsburgh School of Medicine, Pittsburgh, PA 15260, USA; 3 Harvard Medical School Department of Genetics, Boston, MA 02115, USA

**Keywords:** Kidney, Regeneration, Adult organ stem cell, Nephron, Wnt, Frizzled

## Abstract

Zebrafish kidneys use resident kidney stem cells to replace damaged tubules with new nephrons: the filtration units of the kidney. What stimulates kidney progenitor cells to form new nephrons is not known. Here, we show that *wnt9a* and *wnt9b* are induced in the injured kidney at sites where *frizzled9b*- and *lef1*-expressing progenitor cells form new nephrons. New nephron aggregates are patterned by Wnt signaling, with high canonical Wnt-signaling cells forming a single cell thick rosette that demarcates: domains of cell proliferation in the elongating nephron; and tubule fusion where the new nephron plumbs into the distal tubule and establishes blood filtrate drainage. Pharmacological blockade of canonical Wnt signaling inhibited new nephron formation after injury by inhibiting cell proliferation, and resulted in loss of polarized rosette structures in the aggregates. Mutation in *frizzled9b* reduced total kidney nephron number, caused defects in tubule morphology and reduced regeneration of new nephrons after injury. Our results demonstrate an essential role for Wnt/frizzled signaling in adult zebrafish kidney development and regeneration, highlighting conserved mechanisms underlying both mammalian kidney development and kidney stem cell-directed neonephrogenesis in zebrafish.

## Abstract (precis)

**Summary:** Adult zebrafish kidneys induce Wnt signaling to generate new nephrons from resident kidney progenitor cells, highlighting how embryonic morphogens are reactivated in adult organs to drive regeneration.

## INTRODUCTION

Kidney injury and regeneration in mammals occurs by de-differentiation and proliferation of tubular epithelium (Cirio et al., 2013; Guo and Cantley, 2010; Humphreys et al., 2011, 2008). In contrast, adult fish can form entirely new nephrons from resident adult kidney progenitor cells in response to injury (Elger et al., 2003; Reimschuessel, 2001; Zhou et al., 2010). Following intraperitoneal injection of the nephrotoxin gentamicin and damage to proximal tubules (Diep et al., 2011), new nephron formation or neo-nephrogenesis is characterized by the appearance of small aggregates of nephron progenitor cells that form in close association with existing tubular epithelium (Reimschuessel, 2001). Progenitor cell aggregates proliferate, elongate and undergo differentiation to form new nephrons with a functional glomerulus and output connection to the pre-existing tubule architecture (Diep et al., 2011). Transgenic reporter lines have shown that *wt1b* and *lhx1a* promoter transgenes mark new nephrons (Swanhart et al., 2010; Zhou et al., 2010). Specifically, *Tg(lhx1a:GFP)* marks transplantable aggregates of nephron progenitor cells in the adult zebrafish kidney that form functional new nephrons *de novo* in recipient host fish (Diep et al., 2011). Although neo-nephrogenesis has been observed in multiple fish and reptile species (Camarata et al., 2016), and quiescent kidney stem cells are thought to exist in zebrafish (Diep et al., 2011), signaling mechanisms that activate kidney stem cells in response to injury and drive new nephron formation are not known.

Genetic studies in mice have shown that Wnt signaling plays diverse roles during kidney development (Halt and Vainio, 2014). Canonical Wnt signaling is required for the initial stages of nephron formation in which *Wnt9b* from the ureteric bud induces *Wnt4* expression in the nephrogenic cap mesenchyme leading to condensation and mesenchyme to epithelium transformation (Carroll et al., 2005; Stark et al., 1994). Non-canonical Wnt signaling via *Wnt9b* plays additional roles in convergent extension and tubular morphogenesis (Karner et al., 2009; Lienkamp et al., 2012), as well as in regulating self-renewal and differentiation in nephron progenitors (Karner et al., 2011). Loss of β-catenin function specifically in cap mesenchyme blocks nephron induction, while constitutive activation of β-catenin is associated with ectopic mesenchymal condensation and premature depletion of the progenitor population combined with inhibition of mesenchyme to epithelium transformation and loss of further differentiation, indicating that dynamic regulation of canonical Wnt signaling is required for proper nephron development (Park et al., 2007). Other roles for Wnt signaling include a requirement for *Wnt11* in branching morphogenesis and for *Wnt7b* in medullary development (Majumdar et al., 2003; Yu et al., 2009). Currently, less is known about the role of Frizzled Wnt receptors in kidney development. In mice, a double knockout of *Fzd4* and *Fzd8* results in reduced epithelial growth and renal hypoplasia, indicating there may be redundancies in Frizzled function (Ye et al., 2011).

Here, we show that both newly forming and regenerating nephrons in the zebrafish kidney express the Wnt receptor *fzd9b* and the canonical Wnt target gene *lef1*, while the distal tubule and collecting duct are the sites of new nephron formation that express the Wnt ligands *wnt9a* and *wnt9b*. Expression of *fzd9b* mRNA colocalizes with *Tg(lhx1a:GFP)*, a transgenic marker of nephron progenitor cells, and marks new nephron cell aggregates as well as a population of single interstitial cells. Using a combination of Wnt signaling reporters, chemical inhibition and CRISPR/Cas9 gene knockout approaches, our results demonstrate multiple roles for Wnt signaling in adult zebrafish kidney development and regeneration, and suggest that embryonic kidney inductive factors can be employed more broadly to drive kidney regeneration.

## RESULTS

### *fzd9b* and *lef1* expression marks newly forming kidney nephrons

**Fig. 1.**
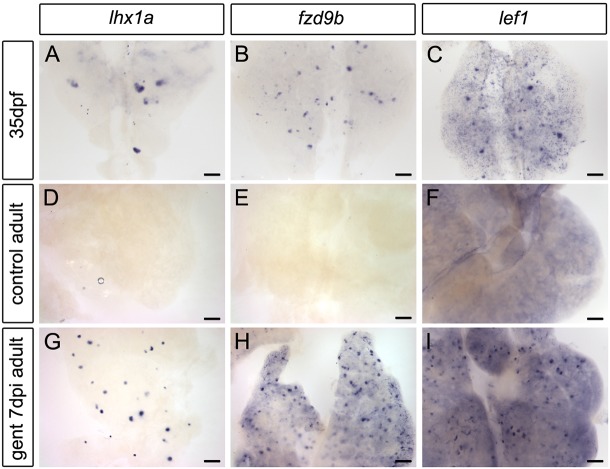
**Expression of Wnt signaling pathway genes during zebrafish mesonephric kidney development and adult regeneration.** (A-C) Whole-mount *in situ* hybridization showing the trunk kidney region of juvenile zebrafish at 35 days post fertilization (dpf). Juvenile zebrafish are rapidly growing and adding new nephrons. The transcription factor *lhx1a* is expressed in cell aggregates comprising newly forming nephrons (A), while the canonical Wnt receptor *fzd9b* (B) and canonical Wnt transcription factor *lef1* (C) appear in similar aggregates, as well as in smaller clusters and single cells. (D-I) Whole-mount *in situ* hybridization showing the anterior trunk kidney region of adult zebrafish (between 6 months and 2 years). (D-F) Adult zebrafish are no longer actively forming new nephrons and only rarely express *lhx1a*, *fzd9b* or *lef1* in aggregates. (G-I) In response to acute kidney injury by gentamicin injection, nephron formation is reinitiated by 7 days post-injury (dpi), and *lhx1a*, *fzd9b* and *lef1* are strongly expressed in large aggregates and small clusters of cells. Representative images from *n*=12 (*lhx1a*), *n*=9 (*fzd9b*) and *n*=10 (*lef1*) juveniles, and *n*=3 (uninj *lhx1a*), *n*=13 (7 dpi *lhx1a*), *n*=3 (uninj *fzd9b*), *n*=5 (7 dpi *fzd9b*), *n*=3 (uninj *lef1*) and *n*=5 (7 dpi *lef1*) adult fish per condition from two independent experiments. Scale bars: 0.1 mm in A-C; 0.2 mm in D-I.

**Fig. 2.**
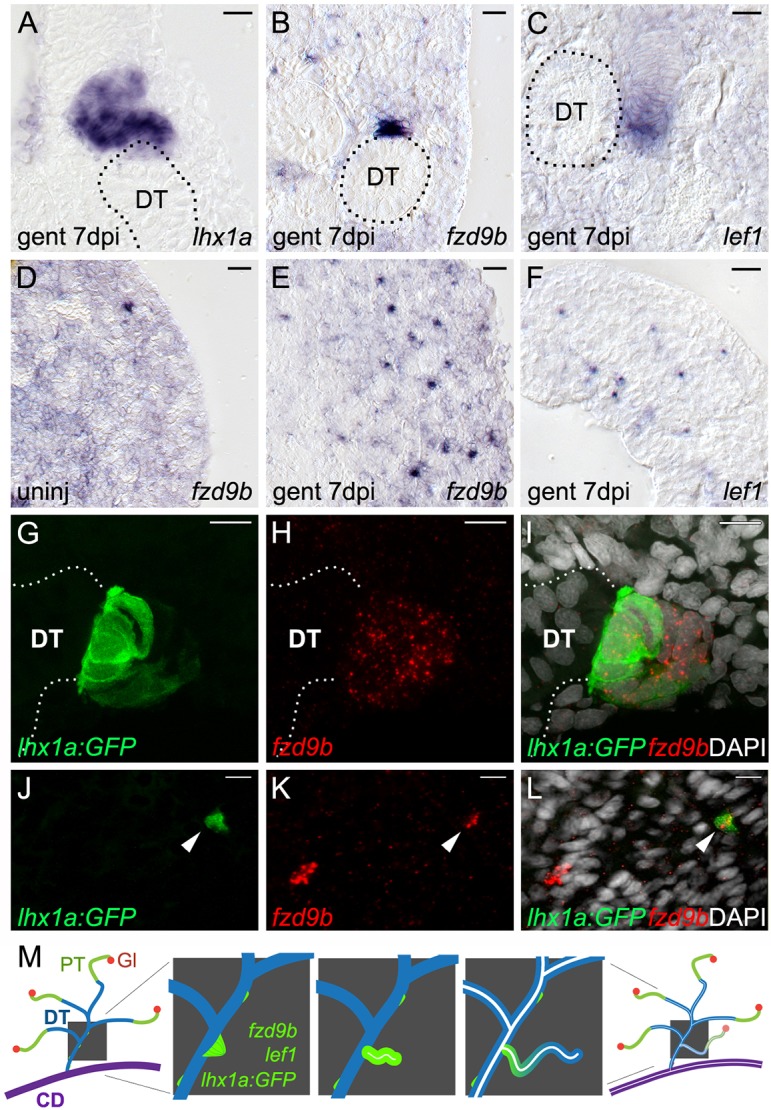
***fzd9b* and *lef1* are expressed in regions overlapping the known nephron progenitor *lhx1a*.** (A-F) High-magnification DIC images of sections from whole-mount *in situ* hybridized kidneys at the indicated time points. (A) The known nephron progenitor marker *lhx1a* is expressed in a new nephron extending from the junction with a pre-existing distal tubule (DT, outline). (B) *fzd9b* is restricted to aggregates and rosettes adjacent to existing distal tubules (DT, outline), while (C) *lef1* extends along the distal end of new nephrons similarly to *lhx1a*. (D) *fzd9b* is expressed in rare single mesenchymal cells in the cortical interstitium of uninjured adult zebrafish kidneys and this population is increased after injury (E). (F) *lef1* is also expressed in many single interstitial cells after injury. (G-L) Confocal images of immunostained whole-mount kidneys from the *Tg(lhx1a:GFP)* reporter line in which nephron progenitors are marked by GFP expression. Fluorescent *in situ* hybridization for *fzd9b* in red and cell nuclei are visualized with DAPI in white. (G-I) 7 days after gentamicin injury, *lhx1a^+^* aggregates marked by GFP also express *fzd9b*. Confocal projection showing a rosette structure in contact with the existing distal tubule (white dotted line). (J-L) Confocal slice showing that single mesenchymal *lhx1a^+^* cells (arrowheads) also express *fzd9b.* (M) Diagram showing stages of new nephron formation from adult kidney stem cells. The adult mesonephric kidney structure showing glomeruli (Gl; red), proximal tubule (PT; green), distal tubule (DT; blue) and collecting duct (CD; purple). Enlarged view of distal tubules (blue) shows sequential steps of new nephron formation initiated with the formation of a polarized rosette of aggregated renal stem cells (green), outgrowth of progenitors to form a primitive tubule, lumen fusion and differentiation of the new nephron (leading to nephron segmentation), and finally development of a filtering fully functional nephron (Diep et al., 2011). Scale bars: 10 µm.

In zebrafish mesonephric development and the regenerating adult kidney, new nephrons are marked by expression of the *lhx1a* gene (Fig. 1A,G) (Diep et al., 2011). In an *in situ* hybridization screen of all zebrafish frizzled genes (*fzd1*, *fzd2*, *fzd3a*, *fzd3b*, *fzd4*, *fzd5*, *fzd6*, *fzd7a*, *fzd7b*, *fzd8a*, *fzd8b*, *fzd8c*, *fzd9a*, *fzd9b* and *fzd10*) and additional markers expressed in similar structures, we found *fzd9b* (a canonical Wnt receptor) and *lef1* (a target of canonical Wnt activity) were expressed in cell aggregates similar to *lhx1a^+^* new nephrons (Fig. 1B,C). *lef1* was expressed in a similar pattern to *fzd9b* but with the addition of low level expression in the interstitium and a discrete salt-and-pepper pattern in tubules (Fig. 1C). Adult zebrafish (older than 6 months) no longer actively producing new nephrons did not show *lhx1a^+^* or *fzd9b^+^* cell aggregates and *lef1* expression was restricted to the interstitial stroma (Fig. 1D-F). Intraperitoneal injection of the nephrotoxin gentamicin results in acute kidney injury and a regeneration response characterized by synchronized production of new nephrons (Reimschuessel, 2001). *fzd9b*, *lef1* and *lhx1a* were all induced in aggregates by 7 days post-injury (dpi) (Fig. 1G-I). Aggregates varied in size, with larger aggregates typically seated on existing tubules and exhibiting a characteristic domed shape with flattened stacks of cells extending away into the newly forming nephron (Fig. 2A-C). Although *lhx1a* was expressed in cell aggregates as well as elongating nephrons, *fzd9b* and *lef1* expression was restricted to cell aggregates and did not appear in more mature new nephron structures (Fig. 2B,C). *fzd9b* and *lef1* were also expressed in single cells scattered throughout cortical areas of the kidney in both uninjured (Fig. 2D) and injured kidneys (Fig. 2E,F). An increase in *fzd9b*-expressing single cells was observed in kidney sections after gentamicin injury, suggesting an expansion of this single cell population in response to nephron damage (Fig. 2D,E). Previous studies have shown that the *Tg(lhx1a:GFP)* transgenic marks single cells and transplantable nephron progenitor cell aggregates in the adult zebrafish kidney (Diep et al., 2011) although we note that this expression differs from the endogenous *lhx1a* gene, which is expressed throughout elongating new nephron tubules and not in single cells (Fig. 2A, Fig. S3A). To determine whether *fzd9b* was expressed in the nephron progenitor cells, we compared GFP localization in the *Tg(lhx1a:GFP)* reporter line (Diep et al., 2011) with *fzd9b* mRNA expression. *fzd9b* mRNA colocalized in GFP^+^ aggregates seated on existing tubules (Fig. 2G-I) as well as with some GFP^+^ single interstitial cells (Fig. 2J-L), confirming that *fzd9b* was expressed in nephron progenitors. Taken together, these results suggest a role for canonical Wnt signaling activity in *lhx1a^+^* nephron progenitors in nephron formation during mesonephric development and injury-induced regeneration. An overview of baseline and regenerative kidney anatomy is shown in Fig. 2M.

### Kidney injury induces  *wnt9a* and *wnt9b* expression in distal tubules and collecting ducts

**Fig. 3.**
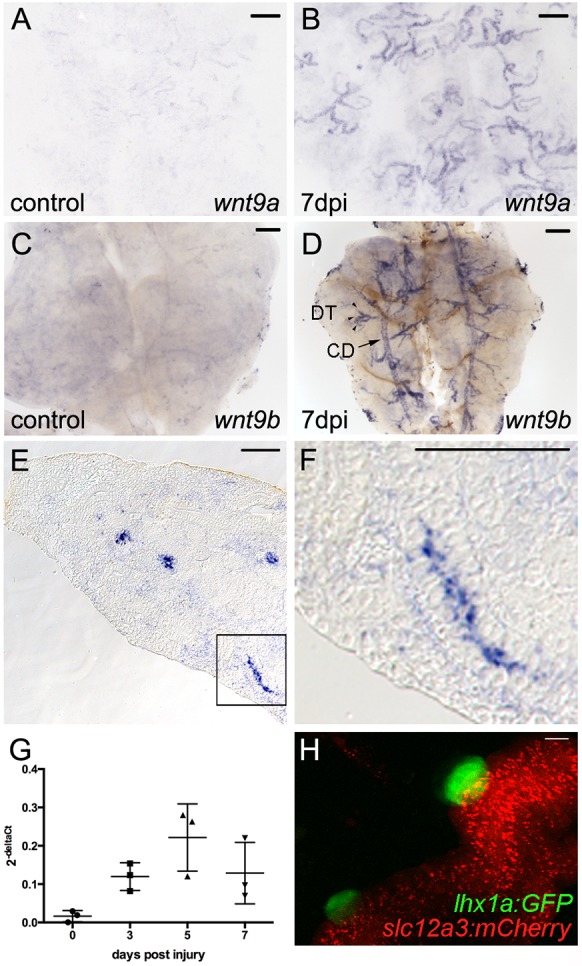
***wnt9a* and *wnt9b* are induced in distal tubules and collecting ducts after injury.** (A,B) Whole-mount *in situ* hybridization for *wnt9a*. (A) *wnt9a* is expressed at very low levels in uninjured adult kidney tubular epithelium. (B) By 7 dpi, *wnt9a* is strongly induced in the branched distal tubule segments of nephrons. (C) *wnt9b* is expressed at low levels in uninjured adult kidney tubular epithelium. (D) After 7 dpi, *wnt9b* is strongly induced in the branched distal tubule segments of nephrons (DT; arrowheads) and common collecting ducts (CDs; arrow). (E) DIC image of a section through a 7 dpi kidney showing *wnt9b* expression in cross-sections of tubular epithelium. (F) Higher-magnification image of boxed area shown in E with *wnt9b* expression in a lengthwise section of tubule. (G) Quantification by qPCR at the indicated time points after injury shows that *wnt9b* expression was increased by 3 dpi and peaked at 5 dpi. Data derived from three individual fish per time point as indicated by individual graph symbols. Data are mean±s.d. (H) Confocal stack projection of *Tg(lhx1a:GFP)*×*Tg(slc12a3:mCherry)* transgenic kidney tissue*. lhx1a:GFP* nephron aggregates form exclusively on *slc12a3:mCherry*-positive distal tubules. Representative images from *n*=2 (A), *n*=3 (B), *n*=6 (C), *n*=4 (D), *n*=3 [H, *Tg(lhx1a:GFP)*×*Tg(slc12a3:mCherry)*] fish per condition. Scale bars: 0.2 mm in A-F; 10 µm in H.

The presence of *fzd9b^+^/lef1^+^* progenitor cells suggested that Wnt ligands may be locally expressed or induced after injury. We screened candidate canonical Wnt ligands expressed after kidney injury and found that both *wnt9a* and *wnt9b* were strongly induced in zebrafish distal tubules and collecting ducts after gentamicin injury (Fig. 3A-F). Wnt9b is a canonical Wnt ligand essential for mouse kidney development (Carroll et al., 2005). *wnt9b* mRNA was localized to the apical cytoplasmic domain (Fig. 3F), as has been observed for other actively translated epithelial mRNAs (Moor et al., 2017). *wnt9b* expression was induced within 3 days post-injury, peaked at 5 days post-injury (Fig. 3G), when nephron progenitor aggregates start to form (Kamei et al., 2015; Zhou et al., 2010), and remained strongly expressed at 7 days post-injury, when aggregates are undergoing proliferation and elongation (see below). The injury-induced pattern of *wnt9a* and *wnt9b* mRNA indicated a site of expression primarily in the branched distal tubules. To determine whether new nephron formation correlated spatially with *wnt9a* and *wnt9b* expression, we examined *lhx1a:GFP* aggregate formation in the *Tg(slc12a3:mCherry)* line (Sugano et al., 2017) that marks zebrafish adult kidney distal tubules and collecting ducts. *lhx1a:GFP*-positive new nephron aggregates appeared exclusively on *slc12a3:mCherry*-positive distal tubules, indicating a tight spatial link between *wnt9a* and *wnt9b* expression and new nephron formation (Fig. 3F and Fig. S1). Our data, together with previous studies showing *wnt4a* expression in new nephron aggregates (Diep et al., 2011), suggest that Wnt signaling may induce and/or pattern *fzd9b^+^/lef1^+^* nephron progenitor cell aggregates.

### Wnt signaling patterns nephron progenitor cell aggregates

**Fig. 4.**
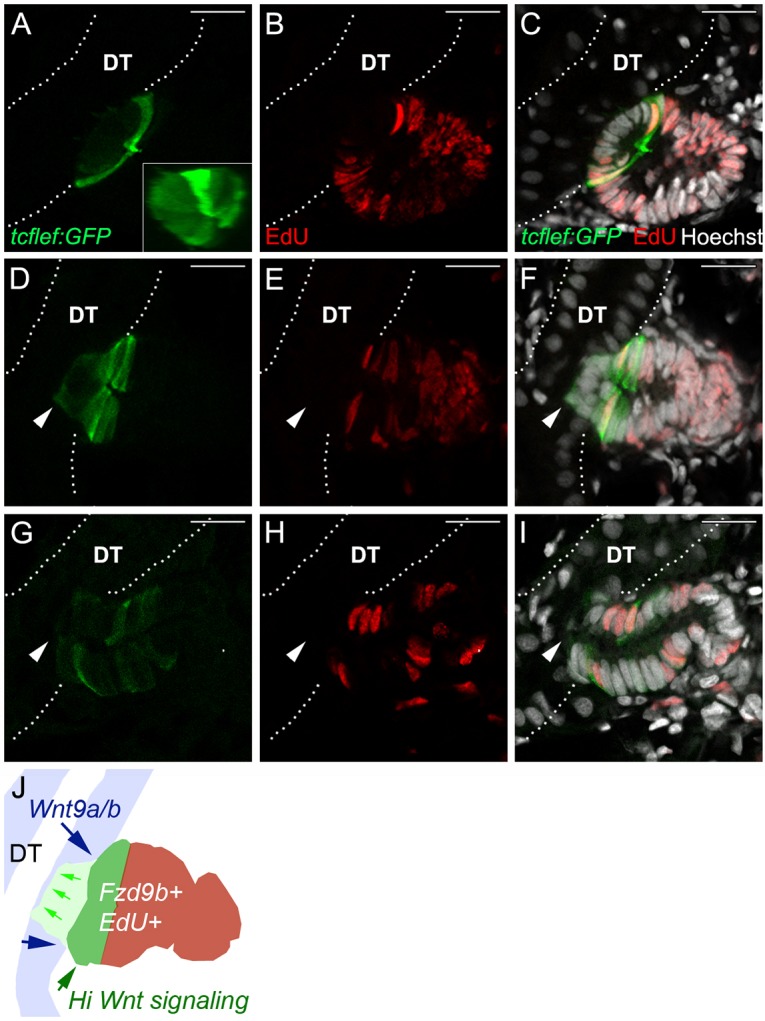
**New nephron aggregates are patterned by canonical Wnt activity that defines a zone of cell proliferation.** (A) *Tg(TCF/Lef-miniP:dGFP)* reporter expression (green) is restricted to a single-cell thick dome of cells, reflecting a domain of high canonical Wnt activity in new nephron aggregates. (B) EdU incorporation (red) in nephron aggregates. (C) Merged image shows cells adjacent to existing tubules are not proliferating, whereas cells in the Wnt^high^ dome and at a distance from existing tubules are highly proliferative. (D-F) An example of a new nephron that has invaded the distal tubule (arrowheads). Invading cells are Wnt^low^, EdU^−^, whereas Wnt^high^, EdU^+^ cells appear flush with the basal surface of the existing distal tubule. (G-I) A new nephron with lumenal connection to the existing distal tubule (arrowheads). Regionalized expression of GFP has been lost and the number of EdU^+^ nuclei has decreased. (J) Diagram of a nephron aggregate at the stage of tubule invasion (F) labeled to show sources of Wnt9a and Wntb (distal tubule, DT; blue, blue arrows), invading cells (green arrows), high canonical Wnt signaling cells (green) and the localization of Frizzled 9b-positive new nephron cells that are also EdU-positive (brown). White dotted lines outline the existing distal tubules (DT). Hoechst labels nuclei. Representative images from *n*=6 fish. Scale bars: 10 µm.

The canonical Wnt reporter line *Tg(TCF/Lef-miniP:dGFP)* (Shimizu et al., 2012) employs TCF/Lef-binding sites in a minimal promoter driving expression of a destabilized GFP to generate a high-resolution view of cells with active canonical Wnt signaling. In injured kidneys, *Tg(TCF/Lef-miniP:dGFP)* was exclusively expressed in a small subset of progenitor cells that marked the distal ends of elongating new nephrons at 7 dpi (Fig. 4A-I) and formed polarized cell rosettes (Fig. 4A-C). Canonical Wnt^high^ rosette structures formed a tight dome of cells with a central apical constriction and with basal surfaces seated on an existing distal tubule (Fig. 4A, inset), similar to the appearance of early lhx1a:GFP-positive new nephron aggregates (Fig. S2). Canonical Wnt^high^ cells were uniformly positive for EdU incorporation, indicating that they were highly proliferative. EdU labeling also revealed that canonical Wnt^high^ rosettes demarcated high and low proliferation regions of the new nephron aggregate. Canonical Wnt^high^, GFP^+^ cells within the rosette and extending away from existing distal tubules were highly proliferative, while cells adjacent to or invading the existing distal tubule were markedly less proliferative (Fig. 4A-C). In new nephrons that had advanced to a lumenal connection with the distal tubule, the discrete dome of GFP^+^ cells was no longer maintained and only scattered proliferating nuclei were observed (Fig. 4G-I). These data demonstrate that canonical Wnt signaling is active and spatially restricted in forming new nephrons and may play a role in patterning proliferation as new nephron tubules elongate. An overview diagram of Wnt sources and Fzd^+^ receiving cells is shown in Fig. 4J.

### Canonical Wnt signaling is required for new nephron formation after injury

**Fig. 5.**
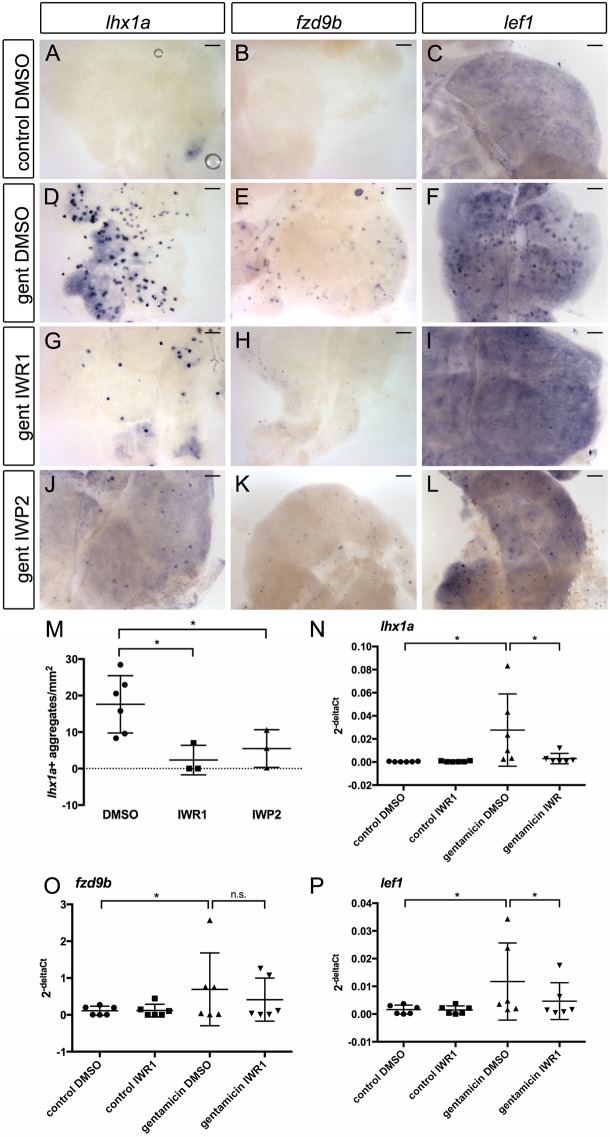
**Wnt inhibition blocks new nephron formation.** Gentamicin-injured adult zebrafish were treated with either DMSO or 5 µM of the Wnt inhibitors IWR1 or IWP2 in system water starting at 1 dpi. Whole-mount *in situ* hybridization showing the trunk kidney region at 7 dpi. (A-C) Control-injected and DMSO-treated kidneys do not express markers of new nephrons. (D-F) Injury induces cell aggregates expressing *lhx1a, fzd9b* and *lef1*. Nephron aggregate formation is blocked by Wnt inhibition using IWR1 (G-I) or IWP2 (J-L). Scale bars: 0.2 mm. (M-P) Quantification of Wnt inhibitor effects on *lhx1a^+^* aggregates and gene expression. (M) Percentage of *lhx1a*^+^ aggregates/mm^2^ of kidney calculated using ImageJ. *n*=3-6 fish for each condition, as indicated by individual graph symbols. (N-P) qPCR quantification of *lhx1a*, *fzd9b* and *lef1* mRNA in kidney tissue harvested 7 d after gentamicin injury, *n*=6 for each condition. *P<0.05 calculated using Student's unpaired two-tailed *t*-test. Data are mean±s.d.

To test whether Wnt signaling was required for new nephron formation or elongation, we assayed the effects of Wnt inhibitors on kidney regeneration. Compared with control kidneys, which showed strong induction of new nephron aggregates at 7 dpi (Fig. 5A-F), injured fish treated with the Wnt inhibitors IWR1 (Fig. 5G-I) or IWP2 (Fig. 5J-L) exhibited significantly fewer *lhx1a*, *fzd9b* and *lef1*-expressing new nephron aggregates. Quantification of these results revealed significant reductions in both the number of *lhx1a^+^* progenitor cell aggregates (Fig. 5M) and the levels of *lhx1a*, *fzd9b* and *lef1* gene expression in gentamicin-injured IWR1-treated fish (Fig. 5N-P). Similar results were observed using XAV939 to inhibit canonical Wnt signaling (Fig. S3).

### Inhibition of canonical Wnt signaling blocks nephron progenitor cell proliferation

**Fig. 6.**
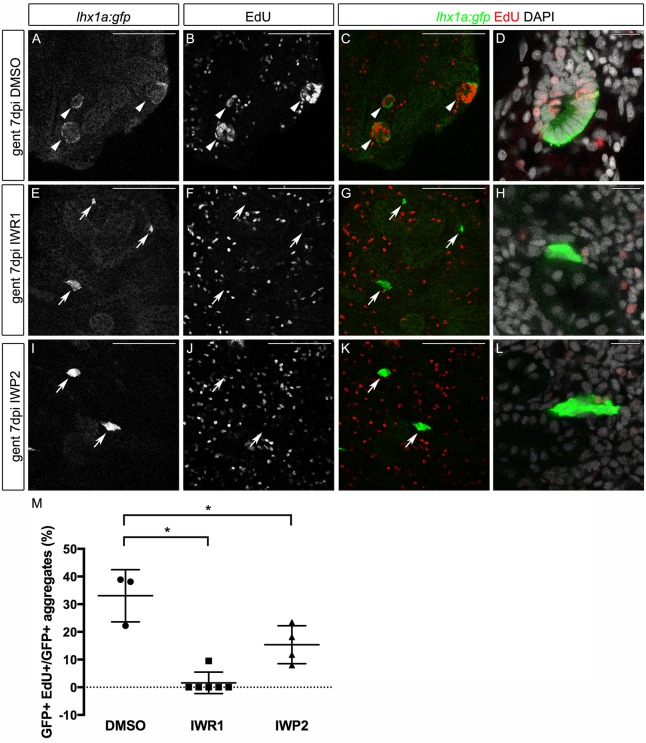
**Wnt inhibition blocks proliferation in nephron aggregates.** *Tg(lhx1a:GFP)* transgenic fish expressing GFP in aggregates and new nephrons were injured by gentamicin injection, injected with EdU to label proliferating nuclei at 6 dpi and kidneys were harvested at 7 dpi. Single slices from confocal *z*-stacks are shown. (A-D) Gentamicin induces GFP^+^ new nephrons with proliferating EdU^+^ nuclei. (E-L) Inhibition of Wnt signaling leads to a loss of proliferation and no morphological sign of nephron formation. GFP^+^ aggregates are still visible adjacent to existing tubules; however, organized structures, such as rosettes or polarized proliferating new nephrons, were rarely observed. Arrows indicate double-labeled new nephrons. Scale bars: 100 µm in A-C,E-G,I-K; 10 µm in D,H,L. (M) Quantification of GFP^+^ aggregates with more than five EdU^+^ nuclei expressed as a percentage of total GFP^+^ aggregates. *n*=3-6 fish for each condition, as indicated by graph symbols. *n*=4-7 confocal *z*-stacks from each kidney. *P<0.05 calculated using Student's unpaired two-tailed *t*-test. Data are mean±s.d.

The association of high canonical Wnt signaling cells with an extending domain of cell proliferation in new nephrons (Fig. 4) suggested that canonical Wnt signaling may be required for nephron progenitor cell proliferation. To test this, we blocked Wnt signaling using IWR1 and IWP2 after injury, and assayed EdU incorporation associated with GFP^+^ aggregates in *Tg(Lhx1a:GFP)* zebrafish. Large EdU^+^ GFP^+^ aggregates were visible in controls at 7 dpi, as elongating immature nephron structures (Fig. 6A-D). Inhibiting canonical Wnt signaling with IWR1 or IWP2 blocked EdU incorporation into GFP^+^ cell aggregates, which remained small and quiescent (Fig. 6E-L), similar to uninjured *Tg(Lhx1a:GFP)* kidneys (Fig. S4). Quantitation revealed that, at 7 dpi, roughly 33% of GFP^+^ aggregates contained more than five EdU^+^ nuclei (Fig. 6M). Inhibition of canonical Wnt signaling significantly decreased proliferation in new nephron aggregates, with only 2% (IWR1) or 15% (IWP2) of aggregates showing 5 or more EdU^+^ nuclei. The spatial restriction of high canonical Wnt signaling to a thin dome of cells observed with the *Tg(TCF/Lef-miniP:dGFP)* transgene (Fig. 4) raised the idea that Wnt^high^ cells could be the direct progenitors for all Edu^+^ cells observed in the new nephrons. However, a short pulse of EdU (2 h) was sufficient to label cells up to ten cells away from the Wnt^high^ domain, revealing that cell proliferation occurred throughout a multicellular domain extending away from distal tubules (Fig. S5), suggesting these cells represent a transit amplifying cell population that generates the new nephron. Cell proliferation (EdU^+^ nuclei) in scattered kidney interstitial cells was not inhibited by IWR1 and IWP2 (Fig. 6E-L), indicating that inhibiting Wnt signaling did not generally suppress cell proliferation in injured kidneys.

### *fzd9b* controls nephron number and morphogenesis following injury

**Fig. 7.**
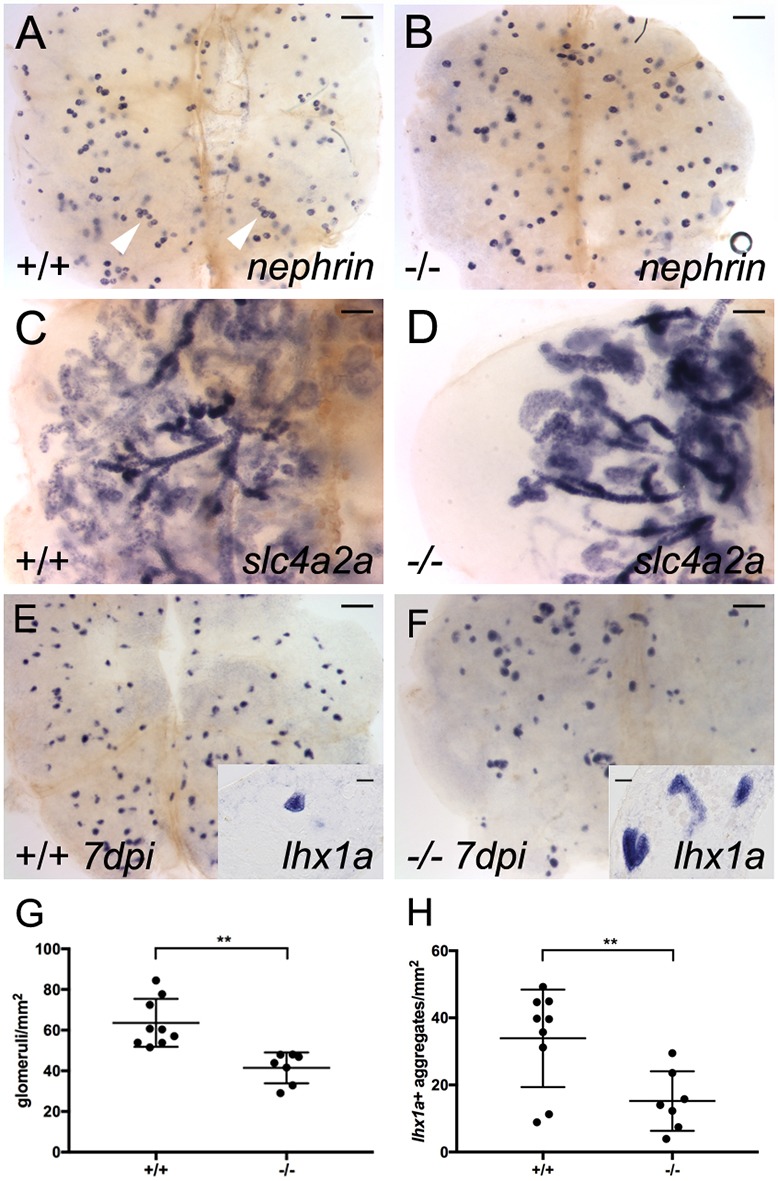
**Mutation in *fzd9b* reduces nephron number and inhibits nephron regeneration.** (A) Whole-mount *in situ* hybridization with a nephrin antisense mRNA probe reveals nephron glomeruli in wild-type adult kidney tissue. Clusters of up to six glomeruli are visible (white arrowheads). (B) In maternal zygotic (MZ) *fzd9b^fb203^* homozygous mutants raised to adulthood, nephrin *in situ* hybridization reveals fewer widely scattered glomeruli in adult kidney tissue (quantified in G). (C) *slc4a2a in situ* hybridization reveals the branched kidney nephron structure in the wild-type adult kidney. (D) In adult MZ *fzd9b^fb203^* homozygous mutants, kidney tissue shows a marked reduction in the number of nephron tubules revealed by *slc4a2a in situ* expression [all *fzd9b*^−/−^ mutants showed a decrease in tubules (*n*=4) compared with unrelated wild type (*n*=3)]. (E) Seven day post-gentamicin injury kidney shows robust production of *lhx1a*-positive new nephron aggregates. (F) MZ *fzd9b^fb203−/−^* adult kidney 7 days post-injury shows markedly reduced *lhx1a*-positive new nephron aggregates. (G) Quantification of nephrin-positive glomeruli per mm^2^ shows a roughly 30% reduction in *fzd9b^fb203^*^−/−^ mutant kidneys. Data represent results from seven to nine individual fish, as indicated by graph symbols. (H) Quantification of *lhx1a*-positive new nephron aggregates 7 days post-injury shows a marked reduction in *fzd9b^fb203−/−^* adult kidney. Student's unpaired two-tailed *t*-test, **P<0.01. Data are mean±s.d. Scale bars: 0.2 mm in A,B,E,F; 0.1 mm in C,D; 10 µm in insets in E,F.

Expression of *fzd9b* during mesonephric development as well as in single kidney progenitor cells and injury-induced new nephron aggregates (Figs 1 and 2) suggested it may play a role in either canonical or non-canonical Wnt signaling during nephron development and kidney regeneration. We generated two CRISPR/Cas9 mutant alleles targeting the *fzd9b*-coding sequence where non-homologous end joining generated a two base pair insertion and a seven base pair deletion, present in both genomic and cDNA sequences, that lead to frame shifts and an out of frame stop codon (Fig. S6). *fzd9b* mRNA was reduced over 1900-fold in *fzd9b^fb203^* and *fzd9b^fb204^* homozygotes, indicating strong nonsense-mediated mRNA decay (Fig. S6D). Adult homozygous *fzd9b* mutants were viable and fertile; however, analysis of both *fzd9b* mutant alleles revealed a reduced complement of nephrons with misshapen tubule dimensions (Fig. 7A-D,G; Fig. S7A,B). Both homozygotes and heterozygotes were affected, with nephron number proportional to *fzd9b* copy number (Fig. S7E). When *fzd9b*^−/−^ homozygous adult mutants were subjected to gentamicin kidney injury, the number of *lhx1a^+^* new nephron aggregates at 7 dpi was dramatically reduced compared with wild type or *fzd9b*^+/−^ heterozygotes (Fig. 7E,F,H; Fig. S7C,D,F). In addition, the *lhx1a*-positive aggregates that did form in *fzd9b* mutants were irregular in size and shape (Fig. 7E,F, insets). The reduced regenerative response was not simply due to a reduction in nephron number (a source of injury-induced Wnt9 ligands) as nephron number was reduced in *fzd9b* mutants by ∼33% (41.46±2.877, *n*=7; *fzd9b* homozygotes versus 63.56±3.926, *n*=9; wild type), while new nephron aggregates were reduced by 55 to 75% (15.21±3.3, *n*=7; *fzd9b^fb203^* homozygotes versus 33.92±4.8, *n*=9; wild type; Fig. 7G,H and Fig. S7C,D,F). In addition, despite nephron morphogenesis defects in *fzd9b*^−/−^ homozygotes (Fig. 7D), there was no quantitative loss of distal tubule marker *slc12a3* expression or decrease in injury-induced *wnt9a* and *wnt9b* expression in *fzd9b* mutants (Fig. S7G-J). The results suggest that Fzd9b is required in kidney progenitor cells to establish proper morphology and number of nephrons.

The distinct phenotypes induced by pharmacological blockade of canonical Wnt signaling (IWR1/XAV939; Fig. 5, Fig. S3) and *fzd9b* mutation (potentially non-canonical Wnt signaling; Fig. 7) suggested that additional Fzd genes might act during kidney regeneration. In single cell RNA seq experiments, we previously identified the Fzd genes *fzd2*, *fzd3a*, *fzd7a*, *fzd7b* and *fzd*10 as being expressed in a putative zebrafish kidney stem cell cluster (Tang et al., 2017). Quantitation of Fzd gene expression revealed that all five of these Fzd genes were upregulated 6.2- to 10.1-fold at 7 days post-injury (Fig. S8). Although the induction was considerably less than the roughly 200-fold induction of *fzd9b* (Fig. S8), it is likely that one or several of these Fzd receptors are also required to mediate Wnt signaling during zebrafish kidney regeneration.

## DISCUSSION

Zebrafish and other cold-blooded vertebrates have remarkable capacities for tissue regeneration (Camarata et al., 2016; Gemberling et al., 2013). In the teleost kidney, neonephrogenesis from adult progenitor cells can replace damaged nephrons (Diep et al., 2011; Elger et al., 2003; Kamei et al., 2015; Reimschuessel, 2001; Zhou et al., 2010); however, the mechanisms underlying progenitor cell-based kidney regeneration have remained unknown. Using genetic and pharmacological approaches to assay signaling pathways activated by kidney injury, we find that Wnt signaling plays multiple roles in adult zebrafish progenitor cell-mediated kidney regeneration. Injury-induced Wnt9b expression in kidney distal tubules precedes formation of *fzd9b*-, *lef1*- and *lhx1a*-positive new nephron cell aggregates that exhibit high canonical Wnt reporter activity. Canonical Wnt signaling is required for cell proliferation in elongating new tubules as a crucial step in nephron formation. On the other hand, mutation in *fzd9b* revealed an additional role for Wnt signaling in regulating the size and number of new nephron cell aggregates. These distinct phenotypes may be rationalized by an interplay of canonical and non-canonical Wnt signaling during nephron formation (Halt and Vainio, 2014; Schnell and Carroll, 2016). Our results are consistent with previous reports that Fzd9 can signal via both canonical and non-canonical pathways (Karasawa et al., 2002), and can bind Wnt9 protein (Matsumoto et al., 2008).

### Wnt signaling is required for nephron elongation

A principal finding of our work is that canonical Wnt signaling is required for cell proliferation and elongation of developing new nephrons after injury in zebrafish. The domain of cell proliferation we observe in new nephron aggregates is conceptually similar to the distal renal vesicle/S-shaped body in mammalian metanephric development (McMahon, 2016). In the mouse, distal renal vesicle cells lie adjacent to the Wnt9b-expressing ureter and express Wnt target genes (Georgas et al., 2009). These cells also define a local domain of high cell proliferation (Georgas et al., 2009) that is likely to play a role in elongation of the distal nephron. Wnt TCFlef reporter transgenes also highlight the S-shaped body as a site of continued high canonical Wnt signaling (Schmidt-Ott et al., 2007). In an analogous fashion, the zebrafish new nephron aggregates abut Wnt9b-expressing distal tubules and report strong canonical Wnt signaling. Alternatively, Wnt signaling could indirectly impact proliferation. Wnt signaling is required in the mouse for coordinated growth of the loop of Henle and formation of the renal pelvis (Yu et al., 2009). In this case, collecting duct Wnt7b expression activates Wnt target genes in neighboring interstitial cells, which are proposed to secrete secondary growth factors that support loop of Henle cell proliferation (Yu et al., 2009). Consistent with this, mutation in the secreted Wnt antagonist Dkk1 results in overgrowth of the renal pelvis, as well as in changes in marker expression in the loop of Henle and collecting ducts (Pietilä et al., 2011). Although more experiments will be required, it is likely that Wnt signaling directly drives cell proliferation in new zebrafish nephrons, given that we observe canonical Wnt signaling reporter activity in these cells. Despite the proximity of new nephron cell aggregates to Wnt9b-expressing distal tubules, no Wnt reporter expression and little proliferation is observed in cells closest to the distal tubule and under the ‘dome’ of high canonical Wnt reporter cells. It may be that the distal tubules also secrete Wnt inhibitors that, together with Wnt9b and other Wnt proteins, pattern the newly forming nephrons.

### A requirement for *fzd9b* in zebrafish nephron formation

Wnt signaling is known to play multiple roles in kidney development (Halt and Vainio, 2014; Schnell and Carroll, 2016). In the mouse, Wnt9b expression drives mesenchyme to epithelial transformation and condensation (Carroll et al., 2005). Wnt9b also acts to balance proliferation and differentiation in the nephron progenitor population (Karner et al., 2011), and later to control spindle orientation and direct nephron elongation (Karner et al., 2009). Wnt4 expression in renal vesicles drives condensation and epithelial polarization (Stark et al., 1994). Additional Wnt ligands and receptors regulate ureter and nephron growth, and cell interactions that drive mammalian kidney morphogenesis (Halt and Vainio, 2014; Majumdar et al., 2003; Park et al., 2007; Tanigawa et al., 2011; Yu et al., 2009). In zebrafish mesonephric development, Wnt9a is expressed in the pronephric distal tubules and nephric duct associated with the formation of *fzd9b*-expressing mesonephric nephron progenitors (Curtin et al., 2011; Diep et al., 2015). Similar to the mouse, early mesonephric nephron aggregates express *wnt4* prior to epithelialization (Diep et al., 2015). Our finding that *fzd9b* mutant zebrafish have approximately half the normal number of mesonephric nephrons indicates a requirement for Wnt signaling in the formation of the zebrafish adult kidney (Diep et al., 2015). Kidney tubules were thicker and had irregular diameters in the *fzd9b*^−/−^ homozygotes, suggesting *fzd9b* signals primarily via the non-canonical Wnt planar cell polarity pathway in the kidney (Karner et al., 2009). Our results are similar to the effect of mouse Wnt9b knockout on tubule convergent extension and non-canonical Wnt planar cell polarity pathways (Karner et al., 2009).

The irregular nephron aggregates that form in *fzd9b* mutants compared with the near-complete loss of proliferating new nephrons after canonical Wnt inhibition indicate that additional Fzd genes function during zebrafish nephrogenesis. In mice, at least two frizzled genes, *Fzd4* and *Fzd8*, contribute to nephrogenesis by promoting ureter growth and branching (Ye et al., 2011). Additional Fzd genes expressed in mouse kidney development include Fzd 2, 6, 7 and 10 (Harding et al., 2011). Our recent single cell RNAseq analysis of the zebrafish kidney (Tang et al., 2017) identified *fzd2*, *fzd3a*, *fzd7a*, *fzd7b* and *fzd10* as additional Fzd genes expressed in a putative *six2a*- and *six2b*-positive kidney stem cell cluster, suggesting these genes may contribute to a network controlling nephrogenesis in zebrafish that is similar to the network in mouse (Ye et al., 2011). Indeed, we observed a 6- to 10-fold upregulation of *fzd2*, *fzd3a*, *fzd7a*, *fzd7b* and *fzd10* after kidney injury. Further work will be required to fully determine the roles of these additional frizzled genes during kidney regeneration.

### Wnt signaling and kidney injury

Our results in zebrafish kidney regeneration parallel studies of injury in mammals where activation of Wnt signaling is observed in several different models of acute renal injury (Zhou et al., 2016). In the mammalian kidney, Wnt signaling is shown to have opposing effects depending on the severity of injury. In mild injury, canonical Wnt signaling supports survival and regrowth of existing tubule epithelial cells to fill gaps where tubule cells are lost to injury (Lin et al., 2010; Terada et al., 2003; Zhou et al., 2012). In prolonged or repetitive injury, paracrine Wnt signaling can activate interstitial cells to transform into fibrogenic myofibroblasts with fibrosis, which leads to chronic kidney disease (Xiao et al., 2016). Together with our work, the results imply that induction of Wnt gene expression and signaling is an evolutionarily conserved response to kidney injury; however, the outcome is defined by the cell context and responses to signaling. Mammals do not maintain a population of Six2-positive renal progenitor cells in the adult kidney (Humphreys et al., 2008; Kobayashi et al., 2008) and so do not maintain the potential to re-initiate nephrogenic responses to Wnt signaling. Instead, fibroblast activation in mammals leads to maladaptive fibrotic responses and chronic kidney disease (Xiao et al., 2016; Zhou et al., 2016), whereas fish do not typically develop fibrosis after kidney injury (Hoppe et al., 2015). While zebrafish exhibit conserved gene pathway activation after injury (Forn-Cuni et al., 2017), the impact of inflammatory cells that may respond to fish kidney injury is currently unknown. Approaches to modulating chronic Wnt signaling after acute kidney injury (Hao et al., 2011) or reintroduction of nephrogenic cells (Bantounas et al., 2018) to regenerate kidney tissue are active areas of investigation in regenerative medicine. Our work using zebrafish as a model of adult kidney regeneration provides a framework for solutions to the problem of restoring injured kidney function.

## MATERIALS AND METHODS

### Fish care and injury protocols

Wild-type TuAB zebrafish were maintained according to established protocols (Westerfield, 2000) and all studies were approved by the MGH IACUC. Each experiment was performed with age-matched siblings reared together to minimize background genetic variation. All adult experiments were performed with fish between 6 and 18 months of age, and maintained at uniform tank density to establish a kidney growth plateau (Kamei et al., 2015). All *Tg(lhx1a:GFP)* fish used in each experiment were heterozygous age-matched siblings from a single homozygous parent outcrossed to wild-type TuAB. Acute kidney injury was induced by intraperitoneal injection of gentamicin as previously described (Kamei et al., 2015). Fish weighed between 0.5 g and 1.5 g. Gentamicin (Sigma) diluted in water or PBS was injected at 80 mg/kg in TuAB and *Tg(TCFLef:dGFP)* fish. Initial *Tg(lhx1a:gfp)* experiments were performed using a 120 mg/kg dose because this line showed resistance to gentamicin. After two generations of crossing to wild-type TuAB in our facility, 80 mg/kg was sufficient to induce injury and therefore this dose was used for later experiments. Drug treatments were carried out with 5 µM IWR1 (Tocris), 5 µM IWP2 (Tocris) or DMSO (Sigma) dissolved in system water starting at 1 dpi until harvesting the kidneys at 7 dpi. Water changes were performed at 3 dpi and 5 dpi by replacing half of the volume with fresh drug-treated water. For proliferation studies, 20 µl of 0.5 mg/ml EdU (Molecular Probes) dissolved in HBSS (Sigma) was delivered by intraperitoneal injection at 6 dpi.

### *In situ* hybridization and immunofluorescence

Whole-mount single *in situ* hybridization was performed as previously described (Thisse et al., 2004) with some modifications (Kamei et al., 2015). Briefly, fish with the head and internal organs removed, leaving the kidneys attached to the dorsal body wall, were fixed overnight with rocking in 4% paraformaldehyde (PFA) (Electron Microscopy Sciences). After washing five times with PBST (phosphate-buffered saline with 0.5% Tween-20), fixed kidneys were removed from the body using forceps and permeabilized with proteinase K (10 µg/ml Roche) in PBST for 1 h with rocking, postfixed in 4% PFA overnight and washed five times with PBST. Zebrafish frizzled cDNAs were obtained from the lab of Dr Jeremy Nathans (Johns Hopkins University School of Medicine, Baltimore, MD, USA) (Wang et al., 1996) and OpenBiosystems with the following accession numbers: *fzd1*, NM_001130614; *fzd2*, NM_131140; *fzd3a*, NM_001042761; *fzd3b*, NM_001080601; *fzd4*, NM_001305469; *fzd5*, NM_131134; *fzd6*, NM_200561; *fzd7a*, NM_131139; *fzd7b*, NM_170763; *fzd8a*, NM_130918; *fzd8b*, NM_131553; *fzd8c*, BC163118; *fzd9a*, XM_003198686; *fzd9b*, NM_131511; and *fzd10*, NM_130917. Probes for *lhx1a*, *fzd9b*, *lef1* and *wnt9b* (Jezewski et al., 2008; Swanhart et al., 2010) were synthesized using DIG RNA labeling mix (Invitrogen). After staining, kidneys were fixed with 4% PFA, cleared with dimethylformamide, depigmented with hydrogen peroxide, transferred into PBS:glycerol (1:1) and imaged on a Leica MZ12 microscope equipped with a Spot Image digital camera. Dehydrated kidneys were embedded in JB-4 plastic resin (Polysciences) and then sectioned at 7 µm using a LEICA RM 2165 rotary microtome and mounted using Permount (Fisher Scientific). Sections were imaged on a Nikon E800 microscope equipped with a Spot Insight CCD digital camera.

Quantification of *lhx1a^+^* aggregates was performed in a blinded manner using ImageJ. Briefly, a single 5× image taken of the widest section of each kidney was thresholded and both area and maximum diameter for each aggregate was counted using the particle analyzer. Total number of aggregates was divided by kidney area measured using the freehand tool to outline kidney tissue in picture. Pictures were relabeled before counting such that the person carrying out the analysis had no knowledge of treatment conditions for each sample.

For immunostaining, the initial fixation step was 3 h instead of overnight but otherwise the same. Kidneys were stained for GFP (1:5000, chick anti-GFP, Torrey Pines; 1:3000, goat anti-chick Alexa488, Molecular Probes). After antibody staining, kidneys were treated for EdU detection (Molecular Probes) according to the manufacturer's instructions with a 1 h incubation and stained with DAPI (1:20,000 Roche) or Hoechst (1:2000 Invitrogen) to detect nuclei. For double *in situ* hybridization and antibody staining, probe was detected using the TSA Plus Cy3 kit (Perkin Elmer) followed by antibody staining for GFP as described above. Stained kidneys were mounted in mounting media (53% benzyl alcohol, 45% glycerol and 2% N-propylgallate) and imaged on a Zeiss LSM5 Pascal confocal microscope with 40× and 63× oil objectives. Images were deconvolved using Huygens Essential and processed using Adobe Photoshop 7.0 software.

### Quantitative RT-PCR

RNA was extracted from kidney tissue using a RNeasy Plus Universal kit (Qiagen), and oligo dT and random primed cDNA were made using the Quantitect Reverse Transcription kit (Qiagen) followed by quantitative PCR (ABI 7500) using Power SYBR Green PCR Master Mix (Applied Biosystems) and primers against *lhx1a*, *fzd9b*, *lef1*, *wnt9a*, *wnt9b*, *slc12a3*, *fzd2*, *fzd3a*, *fzd7a*, *fzd7b* and *fzd10* (Table S1). Gene expression was normalized to *gapdh* mRNA expression and data analyzed using the accompanying software.

### Crispr/Cas9 mutagenesis

One-celled zebrafish embryos were injected with Cas9 mRNA (1.4 ng/embryo) and a gRNA (58 pg/embryo) (5′-CTCTTATGACCTGGAGAGAGG-3′; target underlined) directed against the single *fzd9b* coding exon. Target site DNA oligos were annealed and cloned into the BsaI site of pDR274 (Hwang et al., 2013). Vector linearized with DraI was used as template for Maxi Script (Ambion) T7 RNA polymerase to generate gRNAs that were purified on Qiagen RNAeasy columns. Mutagenesis was verified by sequencing individual injected embryo colony PCR products covering the targeted locus. Founder heterozygotes were isolated by outcrossing grown G0 fish to TuAB wild types and genotyping F1 offspring. Primers for genomic PCR were: forward, 5′-AACAGAGAAGCTGCTCGCGGACT-3′; reverse, 5′-GCTGCTTCCCTCTGATTGTC-3′. Products were sequenced using 5′-GCCAGGAAACTTCACCTTG-3′.

## Supplementary Material

Supplementary information
