# Supplementary material for: Wnt signaling mediates new nephron formation during zebrafish kidney regeneration
Source: Development. 2019 Apr 29;146(8):dev168294. doi: 10.1242/dev.168294 (PMC6503981; doi:10.1242/dev.168294)
Supplement: Supplementary information [file develop-146-168294-s1.pdf]

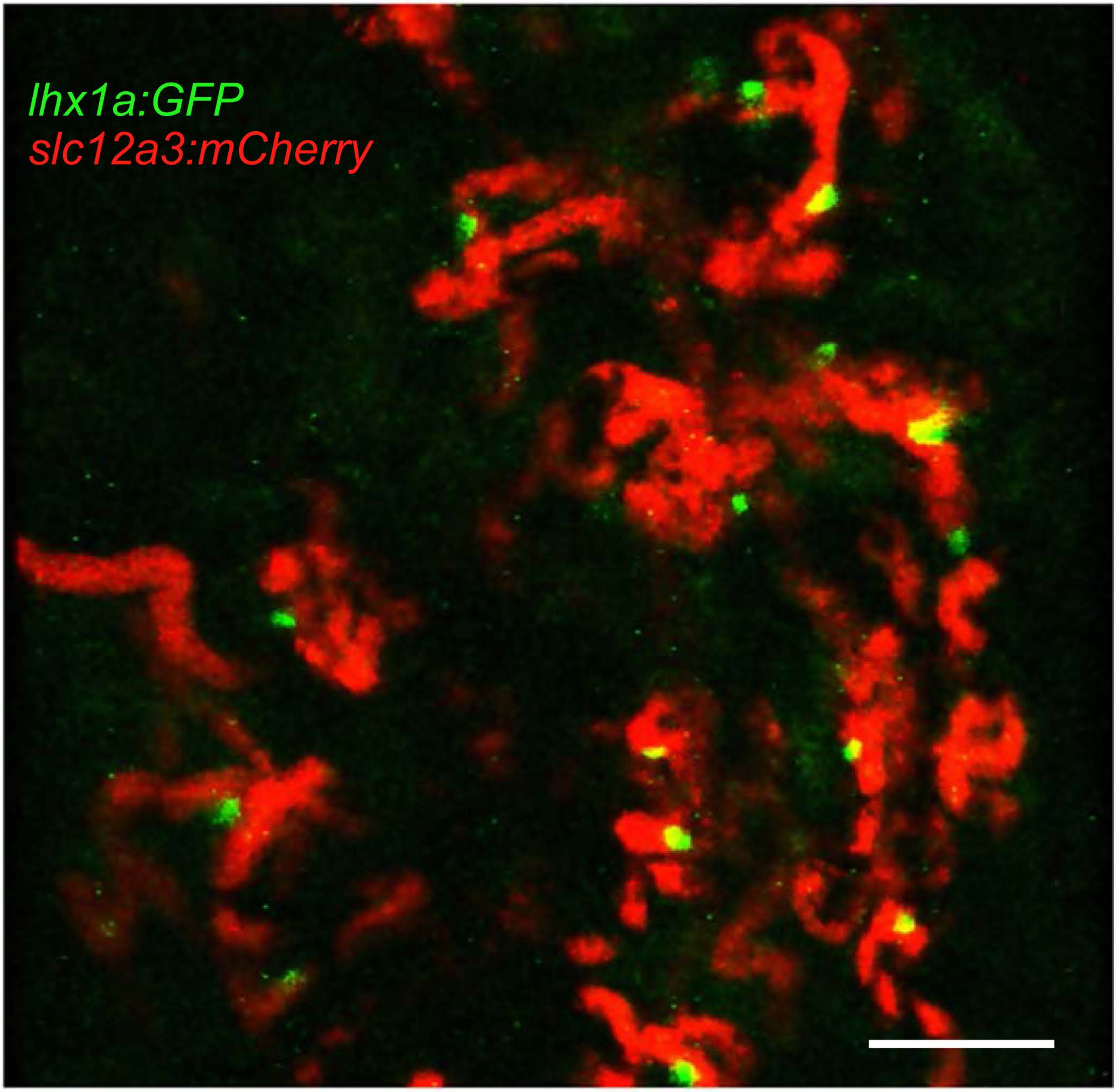

Figure S1. New nephrons form on distal tubules. *Tg(lhx1a:GFP)* positive new nephron aggregates associate exclusively with distal tubules marked with *Tg(slc12a3:mCherry)* in 7 day injured adult zebrafish kidneys. Representative image from n=3 fish. Scale bar = 100 μm.

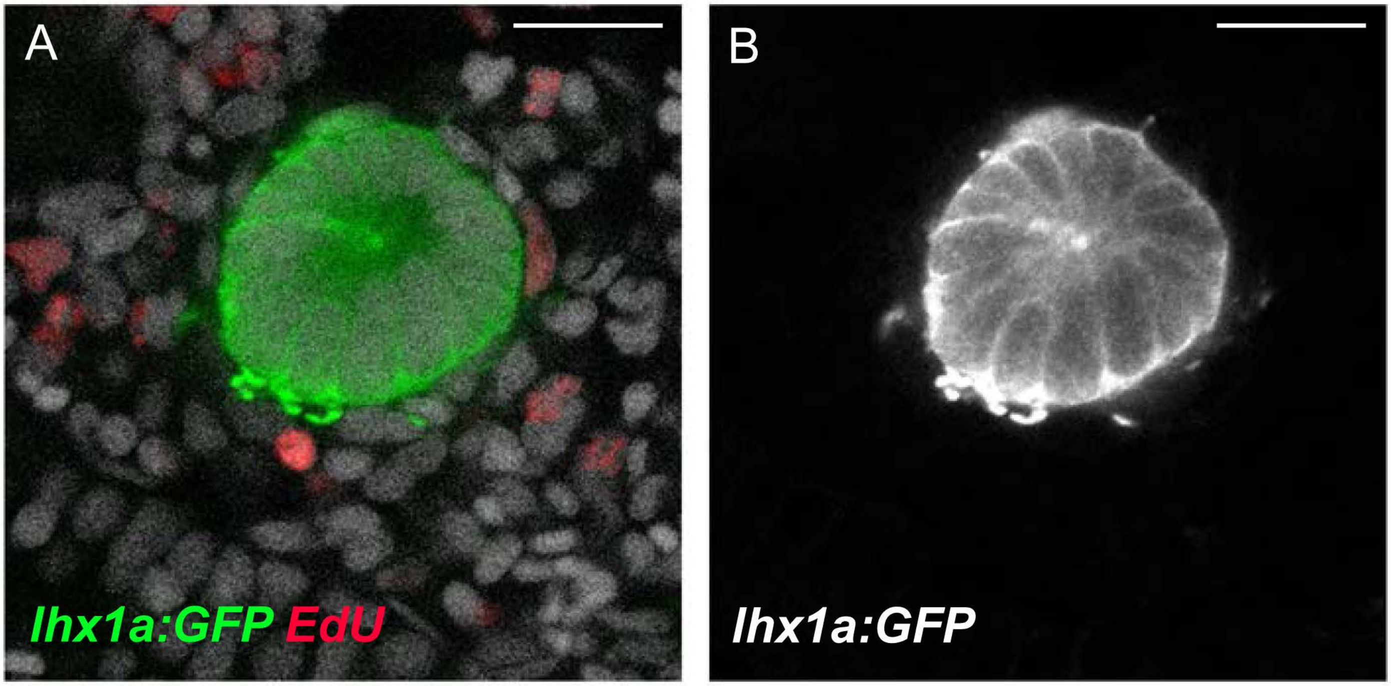

Figure S2. Rosette structure of new nephron aggregates. *Tg(lhx1a:GFP)* transgenic fish expressing GFP in aggregates and new nephrons were injured by gentamicin injection, injected with EdU to label proliferating nuclei at 6dpi and kidneys were harvested at 7dpi and immunostained. (A) Triple labeling with GFP, EdU, and DAPI in a single slice from a confocal Z-stack showing high magnification *en face* view of a GFP+ rosette with characteristic apical constriction and scattered EdU+ proliferating nuclei. (B) High contrast view of GFP fluorescence reveals the cell aggregate rosette structure. Representative image from n=4 fish. Scale bar = 10 μm.

Development • Supplementary information

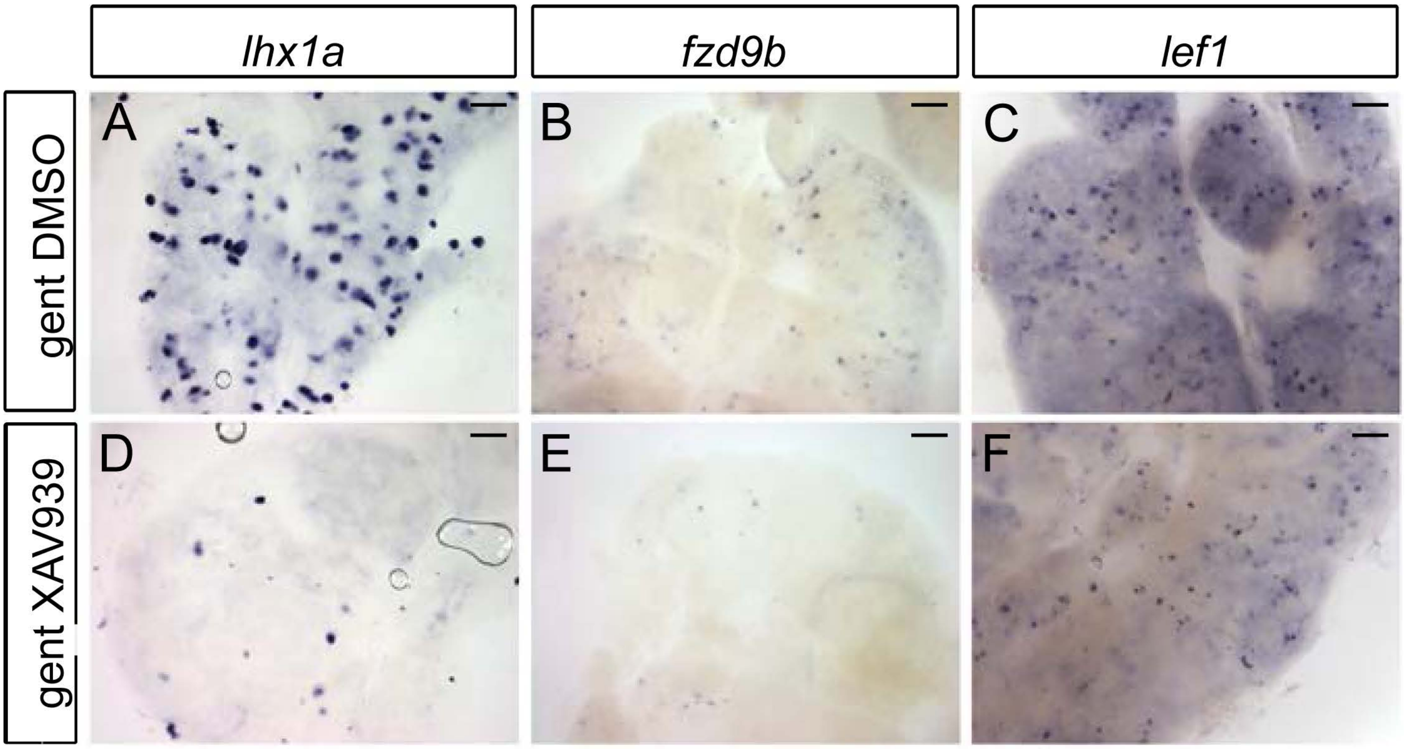

Figure S3. Inhibition of Wnt signaling with XAV939 blocks new nephron formation after injury. Adult zebrafish were injected with PBS or 80 mg/Kg gentamicin and treated with either DMSO or 5 μM of the Wnt inhibitor XAV939 in system water starting at 1dpi. Whole-mount *in situ* hybridization showing the trunk kidney region at 7dpi. (A-C) Injury induces new nephron cell aggregates expressing *lhx1a* (A), *fzd9b* (B) and *lef1* (C). (D-F) New nephron formation is inhibited by the Wnt inhibitor XAV939. Representative images from n=3 (PBS DMSO, PBS XAV) and n=4 (gent DMSO, gent XAV) fish from 2 independent experiments. Scale bar = 0.2 mm.

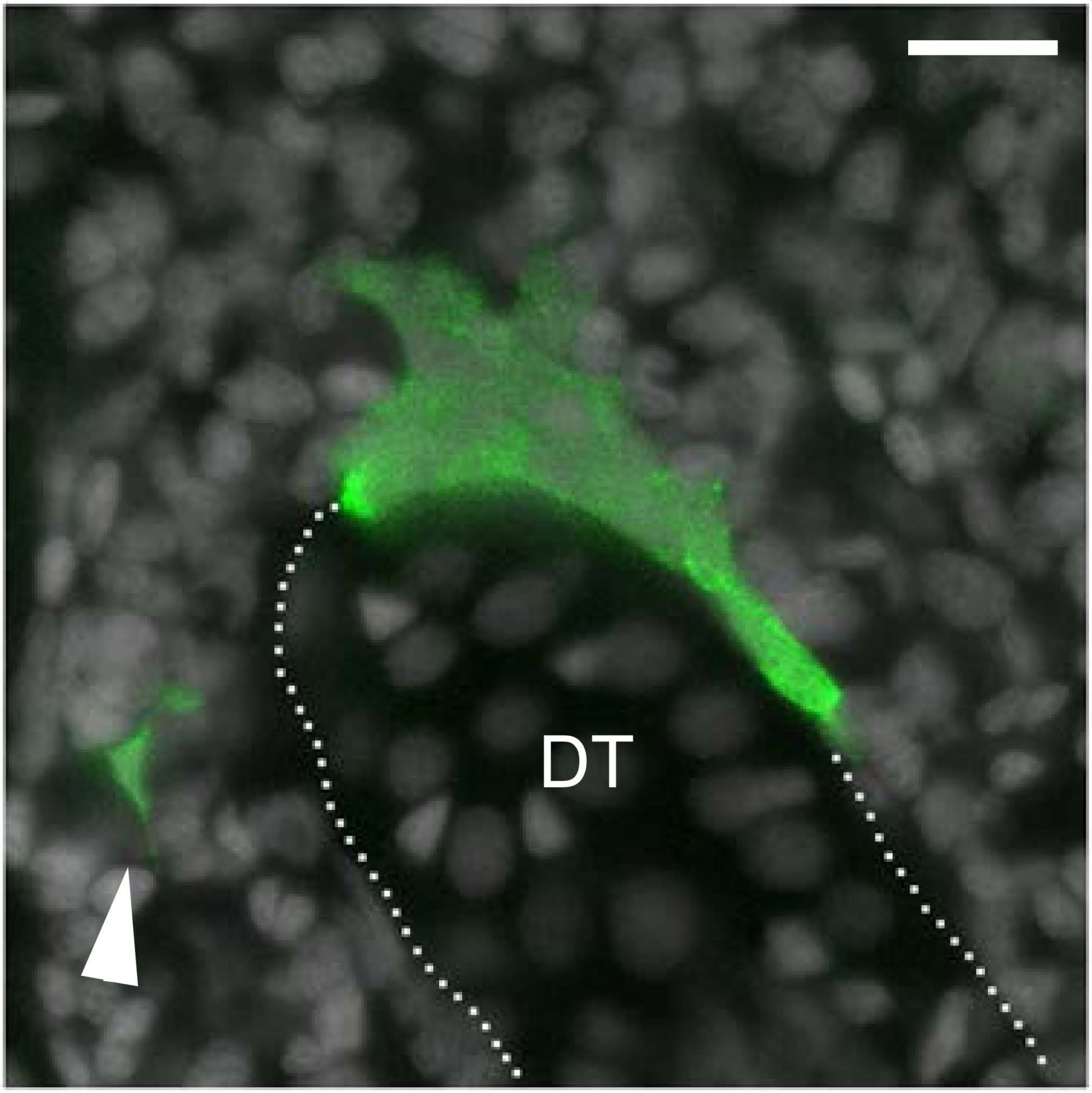

Figure S4. Adult *lhx1a*+ nephron progenitor cells in the uninjured kidney. Stellate morphology of undifferentiated nephron progenitor cell aggregate in uninjured *Tg(lhx1a:GFP)* adult kidney. Representative image from n=3 fish. Scale bar = 10μm.

Development • Supplementary information

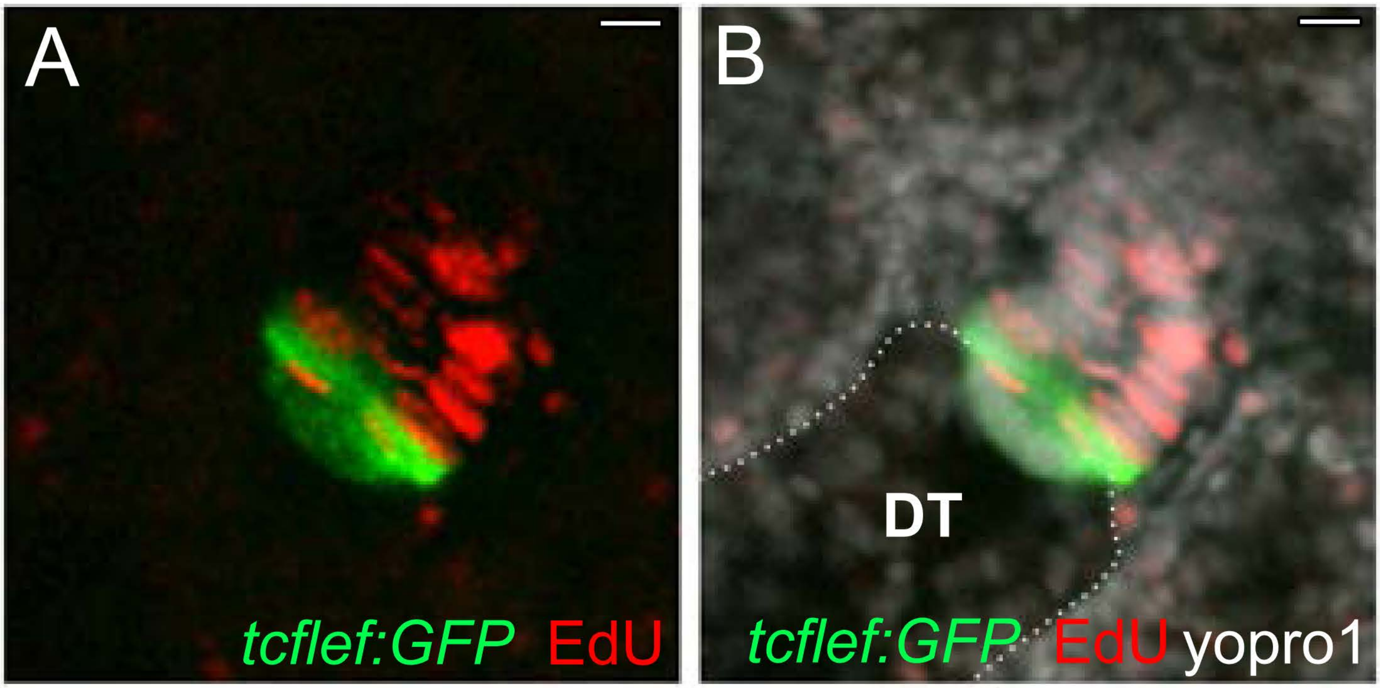

Figure S5. Brief two hour EdU labelling reveals extensive DNA replication in new nephron aggregates. (A) Edu incorporation (red) into *Tg(tcflef:GFP)* positive new nephron aggregates (green) over a two hour period reveals proliferating cells in a broad domain of the new nephron, many cell diameters away from cells with high canonical Wnt activity. (B) Nuclear staining with Yopro1 shows cellular context of the growing new nephron aggregate as it invades the distal tubule epithelium (DT). Representative image from n=3 fish. Scale bars = 10 μm.

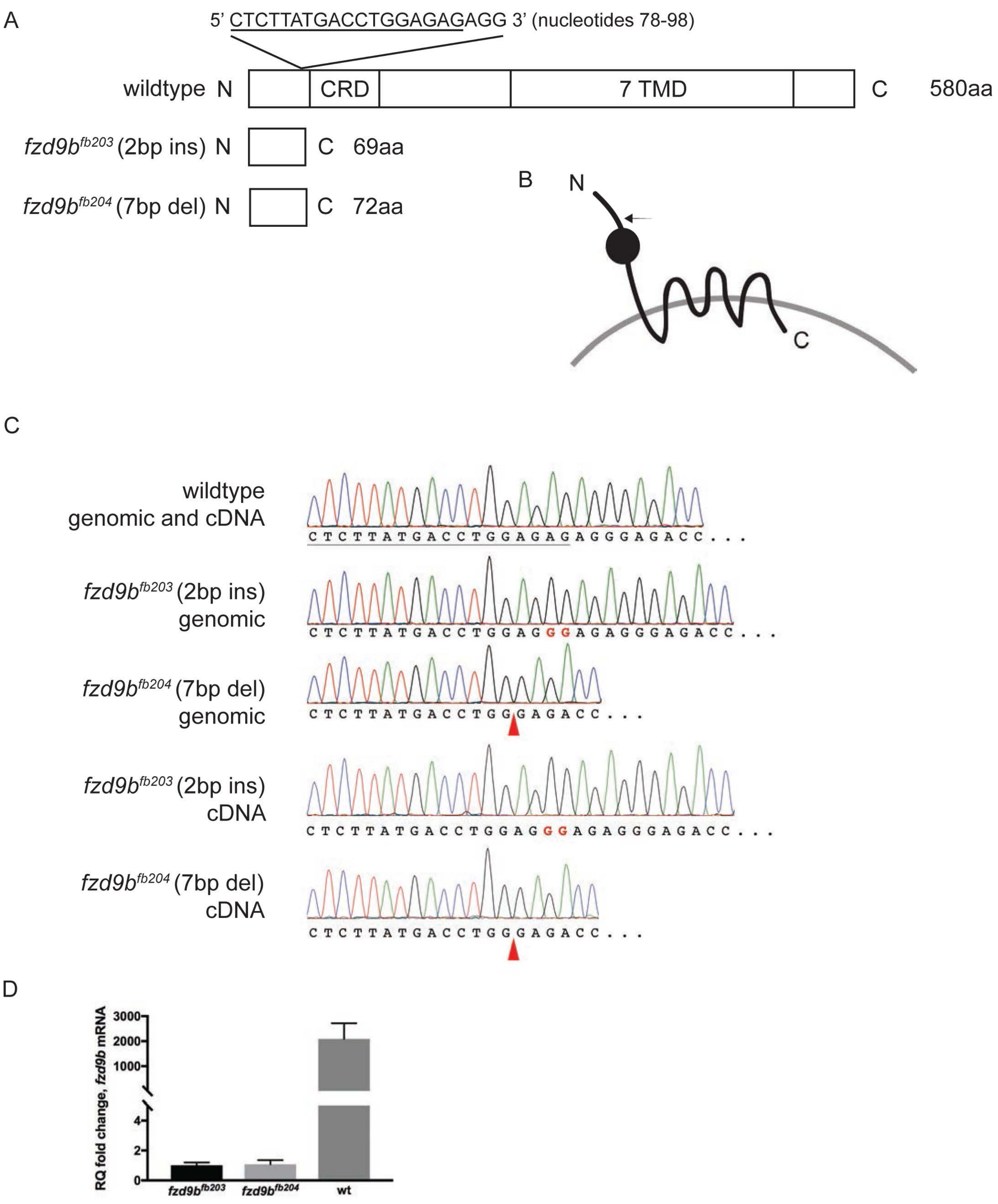

Development • Supplementary information

Fgure S6. Generation of CRISPR/Cas9 *fzd9b* mutant alleles. (A) Targeting site for Crispr/Cas9 mutagenesis of *fzd9b* just N-terminal to sequences encoding the Frizzled9b cysteine-rich domain. Non-homologous end joining repair of Cas9 endonuclease cutting generated two stable *fzd9b* mutant alleles, a 2 bp insertion (*fb203*) and a 7 bp deletion (*fb204*), predicted to encode truncated N-terminal Frizzled9b peptides of 69 and 72 amino acids respectively. (B) Diagram of transmembrane structure of Frizzled9b and mutation site N-terminal to the cysteine rich domain (black circle) in the extracellular Frizzled9b domain. (C) Genomic and cDNA sequence traces of the CRISPR target site in wildtype and *fb203* and *fb204 fzd9b* mutant alleles. (D) Quantitative RTPCR analysis of *fzd9b* mRNA in $fzd9b^{fb203}$ and $fzd9b^{fb204}$ homozygous embryos revealed a greater than 1900 fold reduction in mRNA amount compared to wildtype embryos, indicating nonsense mediated decay. Error bars indicate mean ± s.d.

Development • Supplementary information

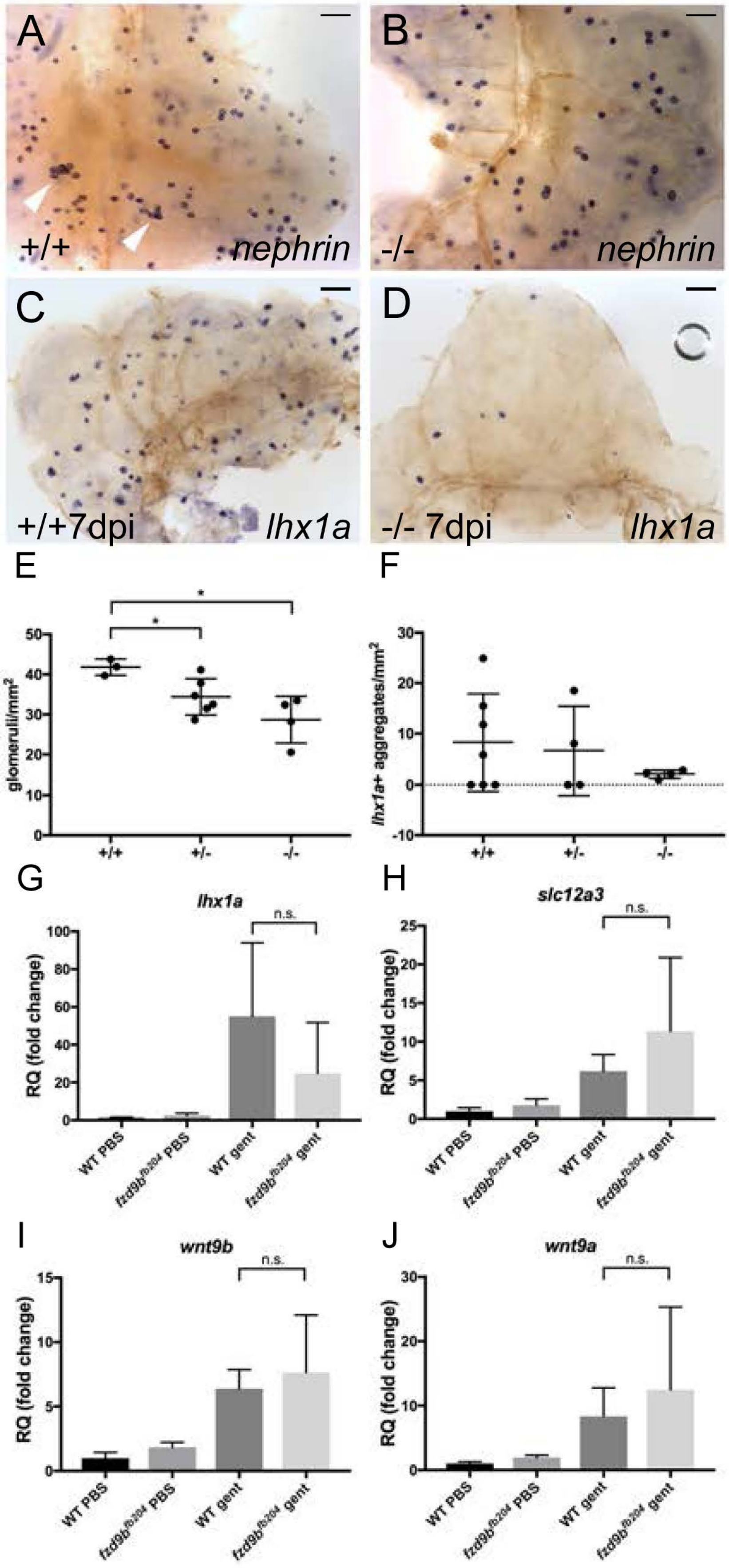
A
+/+
nephrin
B
-/-
nephrin
C
+/+7dpi
lhx1a
D
-/- 7dpi
lhx1a
E
glomeruli/mm2
+/+
+/-
-/-
F
lhx1a+ aggregates/mm2
+/+
+/-
-/-
G
lhx1a
n.s.
RQ (fold change)
WT PBS
fzd9b fb204 PBS
WT gent
fzd9b fb204 gent
H
slc12a3
n.s.
RQ (fold change)
WT PBS
fzd9b fb204 PBS
WT gent
fzd9b fb204 gent
I
wnt9b
n.s.
RQ (fold change)
WT PBS
fzd9b fb204 PBS
WT gent
fzd9b fb204 gent
J
wnt9a
n.s.
RQ (fold change)
WT PBS
fzd9b fb204 PBS
WT gent
fzd9b fb204 gent

Figure S7. Mutation in *frizzled9b* (*fzd9b*^{fb204}) reduces nephron number and blocks kidney regeneration. (A-B) Quantification of nephrons by in situ hybridization for *nephrin* mRNA. (A) Wildtype sibling adult kidney tissue shows more *nephrin*+ glomeruli (arrowheads) than sibling *fzd9b*^{fb204} -/- mutant kidney tissue (B). (C-D) *frizzled9b* is required for nephron regeneration. (C) Wildtype adult kidney tissue seven days following gentamicin injury shows many *lhx1a*+ new nephron aggregates. (D) *frizzled9b* mutant adult kidneys seven days following gentamicin injury show few *lhx1a*+ new nephron aggregates. (E-F) Quantification of *frizzled9b*^{fb204} mutant phenotypes. (E) Glomeruli number (*nephrin*+ glomeruli per mm^2) shows a *frizzled9b* gene dosage-dependent reduction in the uninjured adult kidney. (F) Kidney regeneration (number of *lhx1a*+ new nephron aggregates) is impaired in *frizzled9b* -/- kidneys compared to +/- sibling heterozygotes and wildtype kidneys. (G) RTPCR quantification of *lhx1a* expression in wildtype and *frizzled9b*^{fb204} control and injured kidney mRNA. (H) RTPCR quantification of *slc12a3* expression in wildtype and *frizzled9b*^{fb204} control and injured kidney mRNA. (I) RTPCR quantification of *wnt9b* expression in wildtype and *frizzled9b*^{fb204} control and injured kidney mRNA. (J) RTPCR quantification of *wnt9a* expression in wildtype and *frizzled9b*^{fb204} control and injured kidney mRNA. (G-J) n= 3 (WT PBS), n=4 fish for all other conditions. Student's unpaired two-tailed t-test, * indicates $p<0.05$. Error bars indicate mean ± s.d. Scale bars = 0.2 mm.

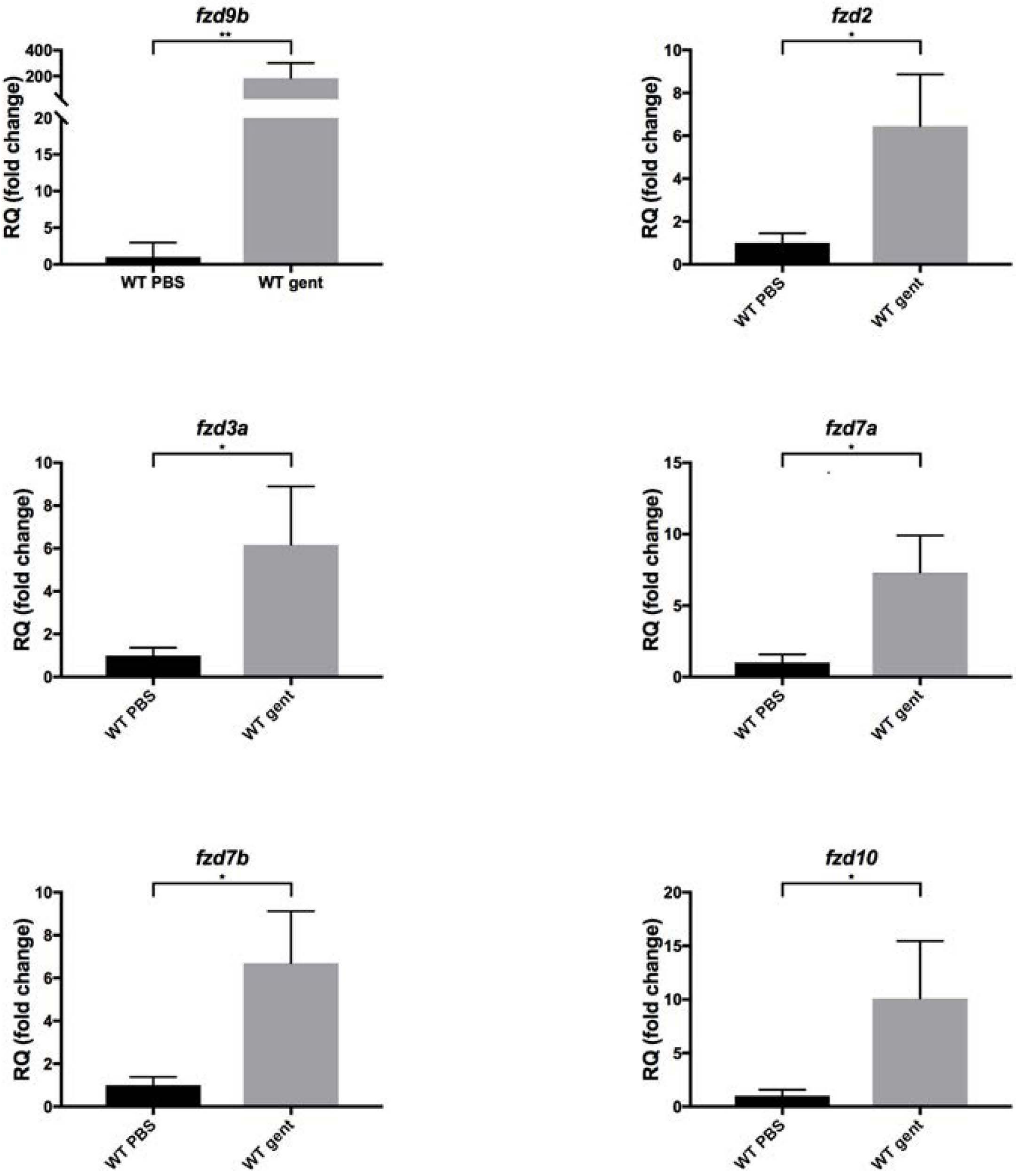
fzd9b
**
RQ (fold change)
400
200
20
15
10
5
0
WT PBS
WT gent
fzd2
*
RQ (fold change)
10
8
6
4
2
0
WT PBS
WT gent
fzd3a
*
RQ (fold change)
10
8
6
4
2
0
WT PBS
WT gent
fzd7a
*
RQ (fold change)
15
10
5
0
WT PBS
WT gent
fzd7b
*
RQ (fold change)
10
8
6
4
2
0
WT PBS
WT gent
fzd10
*
RQ (fold change)
20
15
10
5
0
WT PBS
WT gent

Figure S8. Induction of additional *frizzled* paralog gene expression in the injured kidney. mRNA of *frizzled* paralogs identified in a putative adult kidney stem cell cluster in our single cell RNA seq study (*fzd* 2, 3a, 7a, 7b, and 10; Tang et al., 2017) was quantified by qRTPCR before and 8 days post-injury (dpi). *fzd9b* showed a roughly 200-fold induction by injury while *fzd* 2, 3a, 7a, 7b, and 10 were also significantly induced, suggesting they may play a role in the regenerative response. n= 3 (WT PBS), n=4 (WT gent) fish were used. Student's unpaired two-tailed t-test, ** indicates p<0.01. * indicates p<0.05. Error bars indicate mean ± s.d.

Table S1. Quantitative RT-PCR primer sequences

Gene	Forward (5′ to 3′)	Reverse (5′ to 3′)
gapdh	CGGAGCACCAGGTTGTGTCCA	AGCAATACCAGCACCAGCGTCA
lhx1a	GCTGCGAGTGCAAATGTAAC	CATGCATGTGAAGCAGTTCAG
fzd9b	ATTACGCCTGGCCTGAATCT	GCGCACATGTCTCACTCTTT
lef1	CATCCAGCCATTGTCAACC	CAGCATGAAAGCGTTTAGAGG
wnt9a	AGAATGTGCCGATACAGGGC	AGGTACATCGCTCCATTCGG
wnt9b	TATTGCCCTCTGCATCCTTC	AGAGTCATCTGCTCGCATTG
slc12a3	TGACCAACAACACCTGCACT	TCCTTGCAAAGGCACTGGAA
fzd2	TGGCACCAAGACTGAAAAGC	ATGGCCAGGCTCTTACAGTT
fzd3a	TTGTTAGAGAAGCGGAGGGG	CTGAGCTCCTCCATTCCCTC
fzd7a	TCCGGCTGTTACTTCATGGT	CCTTGCGCGACGGTTTTATA
fzd7b	TCGCCTTGTTGTACGAGTGA	TGTAGACACATGGCCCAACT
fzd10	ACCAGCCATCAAGACCATCA	TGAAGTGCCGATGATGAGGT

Development • Supplementary information
